# Systematic review and meta-analysis of Transurethral Needle Ablation in symptomatic Benign Prostatic Hyperplasia

**DOI:** 10.1186/1471-2490-6-14

**Published:** 2006-06-21

**Authors:** Carmen Bouza, Teresa López, Angeles Magro, Lourdes Navalpotro, José María Amate

**Affiliations:** 1 Agency for Health Technology Assessment, Ministry of Health & Consumers Affairs, Sinesio Delgado 4, 28029 Madrid, Spain

## Abstract

**Background:**

Benign prostatic hyperplasia (BPH) constitutes a major clinical problem. Minimally invasive therapies for the treatment of symptomatic BPH include Transurethral Needle Ablation (TUNA), but it is unclear what impact this technique has on the disease and its role among other currently available therapeutic options. The objective of this study is to ascertain the efficacy and safety of TUNA in the treatment of BPH.

**Methods:**

Systematic review of the literature until January 2005 and meta-analysis of clinical studies assessing TUNA in symptomatic BPH. Studies were critically appraised. Estimates of effect were calculated according to the random-effects model.

**Results:**

35 studies (9 comparative, 26 non-comparative) were included. Although evidence was limited by methodological issues, the analysis of relevant outcomes indicates that while TUNA significantly improves BPH parameters with respect to baseline, it does not reach the same level of efficacy as TURP in respect to all subjective and objective variables. Further, its efficacy declines in the long-term with a rate of secondary-treatment significantly higher than of TURP [OR: 7.44 (2.47, 22.43)]. Conversely, TUNA seems to be a relatively safe technique and shows a lower rate of complications than TURP [OR:0.14 (0.05, 0.14)] with differences being particularly noteworthy in terms of postoperative bleeding and sexual disorders. Likewise, TUNA has fewer anesthetic requirements and generates a shorter hospital stay than TURP [WMD: -1.9 days (-2.75, -1.05)]. Scarce data and lack of replication of comparisons hinder the assessment of TUNA vs. other local therapies. No comparisons with medical treatment were found.

**Conclusion:**

The body of evidence on which TUNA has been introduced into clinical practice is of only moderate-low quality. Available evidence suggest that TUNA is a relatively effective and safe technique that may eventually prove to have a role in selected patients with symptomatic BPH. TUNA significantly improves BPH parameters with respect to baseline values, but it does not reach the same level of efficacy and long-lasting success as TURP. On the other hand, TUNA seems to be superior to TURP in terms of associated morbidity, anesthetic requirements and length of hospital stay. With respect to the role of TUNA vis-à-vis other minimally invasive therapies, the results of this review indicate that there are insufficient data to define this with any degree of accuracy. Overall cost-effectiveness and the role of TUNA versus medical treatment need further evaluation.

## Background

Benign prostatic hyperplasia (BPH), constitutes one of the most common and upsetting disorders among the male population [1-4]. It is associated with lower urinary tract symptoms that interfere with the activities of daily living and sleep patterns, significantly reducing quality of life [2,4] and generating a major burden of sickness and economic cost, both direct and indirect, for society [3,5]. These costs are expected to increase in future due to the longevity of the population and a growing demand for treatment [3,6,7].

The therapeutic options for symptomatic BPH are pharmacological and surgical [1,2,6,7]. Medical treatment often constitutes the initial therapy preferred by patients and health professionals [3,7,8]. However, it is not effective in all patients and some have to stop the treatment owing to the presence of adverse effects [2,7]. Regarding surgical therapies, Transurethral Resection (TURP) constitutes the principal intervention for BPH, to the point where it is now viewed as the standard procedure [2,3,6]. However, TURP is not a suitable form of treatment for some patients, associates with a relatively high morbidity rate and it is not successful in all patients [1,2,9]. Additionally, the high cost generated by this therapy is an important issue for health care providers worldwide [3,5].

In recent years some potentially less invasive procedures such as Transurethral Needle Ablation (TUNA) have become available. The TUNA system consists of a radiofrequency generator, an optical system, and disposable monopolar catheters. The system applies low-level radiofrequency energy (460 kHz) directly to the hyperplastic tissue to produce selective necrosis whilst preserving the urethra and other adjoining structures [10,11]. The procedure was first used in 1993 [10].

TUNA is becoming seen as a possible alternative to conventional treatments -TURP in particular- for symptomatic BPH and is being increasingly used in clinical practice [3]. However, while there are data, including the results of a small-scale recent meta-analysis, which indicate that it may be a good therapeutic option [10,12,13], to date there have been no well-founded conclusions as to its long-term efficacy and safety as stated both by current guidelines on BPH [2,6,14], and the reports of the existing systematic reviews on the topic [15-17], and it is thus still viewed by some authors and institutions as a technique in the research stage [14,16-18].

Accordingly, this study sought was to analyse the collected body of evidence regarding the efficacy and safety of TUNA and its role among currently available therapeutic modalities for the treatment of symptomatic BPH.

## Method

For the identification of studies, we performed a systematic review of the literature [19], without language restrictions, until January 2005 involving the following databases: Medline; CINAHL, CC Search Life/Clin, Pollution & Toxicology, The Cochrane Library, British Library Inside Conference, Serline, Biomedical Journals, Mediconf: Medical Conferences and Events, ISI proceedings. The search strategy has been: #1 (prostat*) #2 (hyperplas*) OR (benign*) OR (BPH), #3 (transurethral needle ablation) OR (TUNA) OR (transurethral) OR (needle) OR (ablat*). Hand searching of the reference lists of included studies and reviews was undertaken and contact was made with experts in the field. However, no contact was made with industry.

### Selection of studies

For inclusion, the studies were required to meet the following criteria [20-22]: a) Design: studies conducted in 10 or more patients that contained relevant primary clinical data; b) Population: patients with symptomatic BPH; c) Intervention: TUNA; d) Comparator: any other medical or surgical procedure; e) Outcomes: the studies were required to include quantitative information relating to at least one of the following primary interest variables: symptom score, quality-of-life score, maximum urinary flow rate (Qmax), post-void residual volume (PVR), pressure-flow studies, need for new therapeutic interventions, adverse effects. The use of health care resources was included as secondary variable. Two independent reviewers carried out study selection and data extraction. A third reviewer checked the resulting extractions and the team resolved any discrepancies.

### Quality assessment

Methodological quality was evaluated for each selected paper using previously validated guidelines [19].

### Data analysis

Intercooled Stata 8 computer software (StataCorp LP Texas USA 1984–2005) was used to obtain an overall measure of the effect of TUNA on the outcomes of interest. Quantitative combination of results was made only when studies showed analogy in terms of type of intervention and clinical homogeneity. We used as a definition of clinical homogeneity that trials have fixed and clearly defined inclusion criteria and fixed and clearly defined outcomes [23]. The meta-analysis was conducted using a random-effects model. Dichotomous outcomes were analyzed using Odds Ratio (OR) or expressed as proportions with their corresponding 95% confidence interval. Continuous variables were analyzed as the difference in means (with 95% confidence interval) between pre- and post-treatment values at the respective assessment dates, with the difference in variances being estimated using standard formulae [24]. In cases where the original study solely furnished the mean of a continuous variable, standard deviation was attributed by weighting the standard deviations of the selfsame variable in other studies [24]. Difference in means was calculated as Weighed Mean Difference (WMD) but when different methods of measurement were used among the studies- such as in symptom scores- we used Standardized Mean Difference (SMD). Statistical heterogeneity was analyzed using the X^2 ^statistic with a P value of < .05. Sensitivity analyses were performed to assess the influence of methodological issues, such as study design, on the overall effect estimation. Absolute Risk Reduction and the Number Needed to Treat (NNT) were estimated where appropriate. The NNT was calculated using the Internet-accessible Visual Rx program . Where it proved impossible for meta-analytic techniques to be implemented owing to the characteristics of the studies or their manner of expressing the results, an individual analysis was performed. Results were calculated on the basis of the respective studies' initial populations, provided that this was possible and a P value of < .05 was deemed significant.

## Results

After eliminating redundancies arising from the use of several databases and studies that failed to report primary clinical outcomes, there were 35 studies that fulfilled the inclusion criteria. From them, 26 were non-comparative and 9 comparative studies. Their respective characteristics, methodological quality and results are outlined separately below. No study that compared the efficacy and safety of TUNA with medical treatment was identified.

### A. Non-comparative studies

Table 1 contains a summary of the 26 studies included. Although a number of efforts were made to prevent any possible duplication of patients, this cannot be entirely ruled in some studies [39,48]. In general, patients were diagnosed as having symptomatic BPH with a symptom duration of over 3 months. Whereas some studies solely included patients with acute refractory urinary retention [26,30,31], other authors [29,47] specifically considered this a criterion of exclusion. In contrast, while median lobe hypertrophy was generally deemed to be an exclusion criterion for TUNA, 2 studies [40,45] typically included this group of patients. Thirteen studies [26,28-30,33,35,36,38,41,43,46,48,49] furnished data on baseline prostatic size, with the mean value being 45.5 ± 8.8 (range:38–66). Only 3 studies [42,44,49] had follow-up periods exceeding 2 years. Only 4 studies [26,33,39,45] expressly stated that industry had been the source, totally or partially, of funding.

In terms of grades of scientific evidence, these were level-IV studies. Even so, study quality, based on the principle of methodologic biases, was acceptable. As shown in Table 1, most were prospective studies, used clear and, in general, homogeneous inclusion and exclusion criteria, plus outcome measures, objective as well as subjective, that were validated and likewise homogeneous. Consequently, selection, performance and detection risks would thus appear to be relatively small. Nevertheless, none contained an express statement to the effect that assessment of results had been performed blindly or independently. Furthermore, despite the fact that some studies reported a scant number of losses to follow-up, in others this number was considerable or unspecified.

#### Efficacy

As Table 2 shows, TUNA led to a significant improvement on both subjective and objective variables over pre-intervention values. Thus, TUNA reduces the symptom index and quality-of-life score by 50%-60% with respect to pre-treatment values, an improvement that is maintained across follow-up, though a progressively downward trend is in evidence after 3 years. With respect to the objective parameters, TUNA leads to improvements which, though significant, amount to an increase of no more than 30–35% over baseline values. Its effect, albeit significant from a statistical point of view, is poorer in term of improvement of urodynamic parameters (Table 2). In some variables (Qmax, PVR) a delayed improvement in outcomes is observed, which in all likelihood is the result of a reduction in the number of patients analyzed and may therefore not signal any new real improvement. Combined analysis of 4 studies [30,33,35,39] showed that in most patients (approximately 80%) there were no notable changes in sexual function following the procedure.

Some authors [28] state that the effect on objective variables is weaker in the case of prostates larger than 50 grams. Others, however, do not observe that size exerts a significant influence on their patients' response and suggest that this should not be an exclusion factor for the use of the system [36,38]. Additionally, the results of five studies [25,26,30,31,47] indicated that 70% of patients (78/112) with acute/chronic urinary retention achieved spontaneous voiding in the first week post-procedure. In like fashion, studies that analyze the effect of TUNA on patients with median lobe enlargement [40,45] report improvements similar to those among the general population with symptomatic BPH

Few studies have endeavored to identify the factors implicated in response to treatment and its results indicate the absence of significant differences between patients who do and do not respond to the treatment, in terms of age, prostatic volume, objective or subjective mean baseline scores, position of TUNA needles, number of lesions, or temperature attained [36].

**Figure 1:**
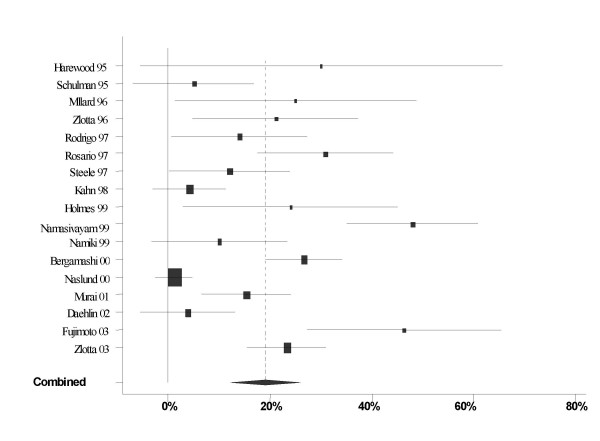
**TUNA. Non-comparative studies. Rate of secondary interventions**. Random effects model.

Seventeen studies [26,28,30,31,34-36,38,41-49] give data on the need for new interventions and, though their individual results varied widely, combined analysis indicates that 237 of 1036 patients treated with TUNA required new treatments, amounting to an overall re-treatment rate of 19.07% (95% CI:18.7–39.7) (Figure 1). These new treatments fundamentally consisted of surgical measures, namely: TURP (150 cases); unspecified surgery (22); second TUNA (7); prostatectomy (6); and transurethral incision of the prostate (1). A total of 41 patients received drug therapy. In 10 cases, the treatment employed was not specified.

#### Safety

The description of adverse effects was heterogeneous and in general ambiguous. Table 3 lists the adverse effects identified in the individual studies. Due to the fact that 4 studies did not include data on safety [10,41,42,49], the percentage calculation was based on 1204 patients included in the 22 studies that did furnish such data.

As shown by this table, the most frequent adverse effect was hematuria, which in most cases took the form of mild bleeding. Overall, the series described a total of 16 cases of severe hematuria, with only one patient requiring transfusion. In contrast, 279 cases of urinary retention were described, which proved transitory in all but two cases. Attention should be drawn to the variability of outcomes in the 18 individual studies reporting this adverse effect, a variability that seems to be due, on the one hand, to the different patient-selection criteria employed and, on the other, to the practice of using or not using routine post-operative catheterization. Also appearing with considerable frequency were dysuria, of lesser or greater intensity; irritative symptoms, which were usually transitory but were not clearly described and; urinary tract infections. With respect to adverse effects on sexual function, Table 3 lists the scant number of cases registered.

Only a very small number of studies mentioned the presence of adverse effects during the procedure. Kahn [38] reported the existence of intense pain during the procedure in 4 patients under the age of 65 years, which caused the TUNA to be halted in two cases, and was resolved by means of perineal block in the other two. This same author described that 10 patients reported a medium-moderate burning sensation, which in two cases made it necessary for the treatment to be halted. Roehrborn [39] stated that 22% of patients analyzed reported pain and discomfort, and Dæhlin [47] observed that 42% of his patients presented with moderate and 4% with major malaise.

#### Use of healthcare resources

In 43.5% of studies the procedure was performed under local anesthesia and conscious sedation with an estimated mean duration of 45 ± 18 minutes. Insofar as hospital stay was concerned, 12 studies furnished quantitative data. In 9 of these, the stay was less than a day, in one it was 1 day, and in two it was more than 2 days. With respect to the number of patients included in these studies, 87% of patients remained in hospital for just a few hours after TUNA had been performed. No study formally analyzed the costs of the procedure.

### B. Comparative studies

All studies compared TUNA with TURP whereas 4 compared TUNA with other minimally invasive techniques such as: Transurethral Microwave Thermotherapy (TUMT), Water-induced Thermotherapy (WIT), Interstitial Laser Coagulation (ILC), Transurethral Electrovaporization (TUVP), Visual Laser Ablation (VLAP) and Transrectal High Intensity Focused Ultrasound (HIFU).

In terms of design, 4 were non-randomized and 5 were randomized studies. Of these, three [50,51,54] were papers based on a single clinical trial. Nevertheless, it was decided that all should be included since they furnished information on different variables and follow-up periods. Bearing this in mind, the number of patients included in the randomized studies was 336 (167 treated with TUNA, 169 with TURP). Their general and methodologic characteristics are shown in Table 4. In line with these characteristics and biases, these studies provide level-II evidence.

In addition, we identified 4 non-randomized studies (level-III evidence) [55-58]. Of the two papers by Schaltz which correspond to a single study, the more complete [57] was used for analysis of efficacy. We also decided to include the 1997 paper [55], since it furnished safety data that were not included in the later paper. The characteristics of these studies are set out in Table 4. Allocation to each treatment arm was made in line with the wishes of the patient [56], sequentially [55,57] or in accordance with anesthetic risk, prostatic volume and the patient's desire to maintain normal ejaculation [58].

### 1. TUNA vs. TURP

#### Clinical efficacy

The results of comparisons are shown in Table 5. Both techniques led to significant improvements in BPH parameters and initially, the efficacy of TUNA would seem to be equivalent to that of TURP. However, at 12 months subjective parameters and objective functional measures are both statistically better in the latter case, with the differences becoming increasingly greater across time. In some variables a delayed uniformity in results is observed, which in all likelihood is the result of a reduction in the number of patients analyzed and may therefore not signal any new real improvement in the group treated by TUNA.

**Figure 2:**
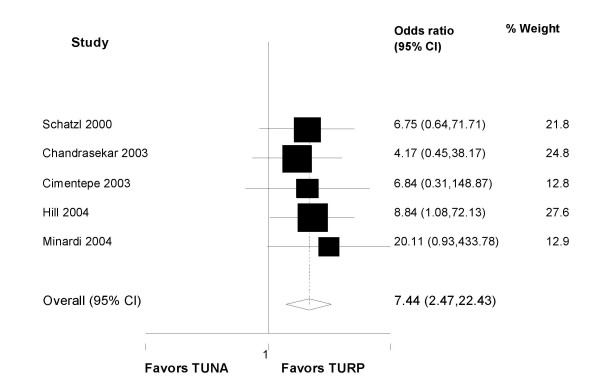
**TUNA vs. TURP. Need for new therapeutic interventions**. Random effects model.  CI denotes confidence interval Chi-squared heterogeneity = 0.70 (d.f. = 4), P = .951. Overall effect P value = .000.

The combined results of the studies that furnished data (Figure 2) indicated that TUNA had a re-treatment rate significantly higher than that of TURP given that 10% (21/206) of TUNA versus a mere 1% (3/282) of TURP patients required new treatments.

The effects of treatment on sexual function were analyzed in 3 studies [50,53,56] that used different scales. In one randomized clinical trial, Bruskewitz [50], using a 7-item questionnaire, observed a clearly different response in the two treatment groups, inasmuch as 53% of the TURP versus only 13% of the TUNA cohort reported diminution in the volume ejaculated (P < .001). Cimentepe [53] observed that 100% of patients treated with TUNA reported no change whereas 61% of patients treated with TURP reported deterioration in sexual function. For his part, Arai [56], in a non-randomized study, observed a mild-moderate deterioration in erectile function in 26.5% of subjects treated with TURP and 20% of subjects treated with TUNA, without there being any significant change in the pre- and post-treatment indices of erectile function or libido in either group. In 48.6% and 24% (P < .001) of TURP and TUNA patients, respectively, there was loss of ejaculation capacity or an appreciable diminution in the volume ejaculated.

#### Safety

**Figure 3:**
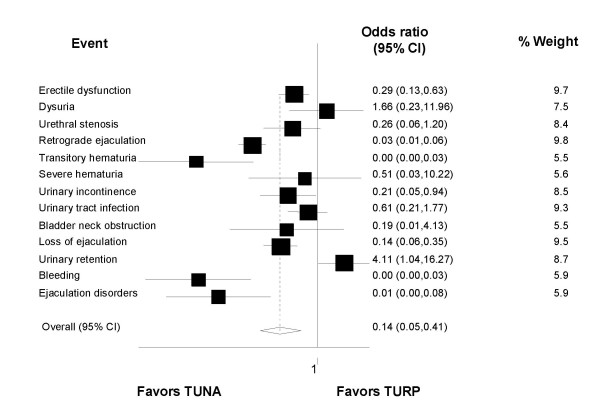
**Studies that compare TUNA and TURP. Adverse effects. Results of meta-analysis**. Random effects model.  CI denotes confidence interval.

There was no mortality associated with either of these procedures whereas a total of 67 and 417 adverse effects were reported for the TUNA and TURP groups respectively. The combined analysis of the results is shown in Figure 3.

In both procedures, irritative symptoms appeared to be frequent and, though somewhat unclearly described, were usually transitory. Only one study [53] referred to their duration, indicating that these remained in evidence for 7–10 days in the case of TUNA and 2–3 weeks in the case of TURP. Apart from such symptoms, the most frequent adverse effects were postoperative bleeding and those linked to sexual dysfunction. Whilst the description of the presence of postoperative bleeding in TURP patients was a constant feature of almost all the studies -with one [52] reporting that up to 10.5% of patients needed transfusion- not a single case of TUNA was cited as requiring transfusion.

Similarly, adverse effects on sexual function were less frequent in the TUNA group. The studies reviewed described 7 cases of urinary retention in the TUNA and 3 in the TURP group, a finding that that favors the latter technique from a statistical point of view. Nevertheless, the difference observed between studies in routine indication and duration of post-operative catheterization, coupled with the poor descriptions given, make this complication difficult to evaluate.

As is graphically depicted in Figure 3, treatment with TUNA resulted in a significantly lower number of complications than TURP [OR: 0.14 (95% CI:.05-.41)]. This amount indicates that, overall, TUNA entailed an Absolute Risk Reduction of complications of 19.4% (95% CI:17%-22%), with an estimated NNT of 5 (95% CI:5–6) to prevent a bad outcome.

#### Use of healthcare resources

In 50% of the studies, TUNA was performed under local anesthesia, with or without associated sedation, whereas TURP was performed in all cases under general or regional anesthesia. Regarding the estimated mean duration of the procedures, the difference was statistically significant in favour of TUNA [WMD: -13.22 minutes (95% CI:-17.8, -8.7), P = .00]. Analysis of the 4 studies [52-54,57] that furnished data indicated a hospital stay almost two days shorter in the TUNA (mean value:1.22 days) than in the TURP group (mean: 2.84 days), with the difference proving statistically significant [WMD: -1.9 (95% CI:-2.75, -1.05), P = .00]. None of the studies conducted any analysis of the costs associated with the respective procedures.

### 2. TUNA vs. other minimally invasive therapies

The results of these comparisons are shown in Tables 6 and 7. Arai's study [56] showed that, in the assessment at 3 months, TUNA-related improvements were significantly superior to those of TUMT in terms of symptom index, quality-of-life score, Qmax and PVR; in addition, there was a trend, albeit non-significant, towards a lower number of erectile dysfunctions.

Likewise, TUNA seemed to have a significantly better effect than WIT on the objective variables analyzed in the study by Minardi [58]. The results vis-à-vis ILC proved more difficult to analyze, as each of the techniques seemed somewhat more effective than the other, depending on the point in time at which they were evaluated. However, adverse effects tended to be more frequent in the group treated with ILC, particularly in the case of irritative symptoms, postoperative hematuria, and reduction in seminal volume (Table 7).

Analysis of the studies that compared TUNA with TVP [55,57,58] indicated that TVP achieved improvements significantly superior to TUNA in both subjective and objective variables, but that this was accompanied by a higher incidence of adverse effects.

As against VLAP, Schatzl's results based on 30 patients indicated that, while both techniques led to similar improvements in the symptom index, VLAP exercised a significantly superior effect on the objective variables (Qmax and PVR). Furthermore, there seemed to be fewer adverse effects with this technique. Notable differences between TUNA and HIFU were not in evidence in the analysis of the 35 subjects in whom these techniques were compared.

## Discussion

This study analyzes the results of 35 primary studies on TUNA in the treatment of BPH and, to our knowledge, represents the most complete systematic review conducted to date, by virtue not only of the number of studies included, but also of the scope of the variables analyzed and the length of time covered by the analysis as compared to previous systematic reviews [15-17]. A total of 26 clinical series and 9 comparative studies assessing TUNA vs. TURP and other minimally invasive techniques such as TUMT, WIT, ILC, TUVP, VLAP and HIFU met the inclusion criteria. No study that compared the efficacy and safety of TUNA with medical treatment was found in literature.

According to the data obtained, TUNA significantly improves BPH parameters with respect to baseline values with improvements being particularly noteworthy in terms of symptomatic alleviation and quality of life scores. In addition, TUNA seems to be a relatively safe technique with minimal effects on sexual function, has few anesthetic requirements, is of short duration and generates a short hospital stay. Against this, the greatest risks posed by the technique lie in its high re-treatment rate given that, in the long-term, a high proportion of patients need a new therapeutic intervention, be it medical or surgical, though the pre- or intra-operative variables associated with this need for re-treatment are as yet unknown.

Comparisons with TURP show that, except in the very short-term when TUNA achieves a similar level of efficacy specially in symptoms and quality of life scores, the degree of improvement in respect of all subjective and objective variables was significantly lower than of TURP across time with differences being particularly noteworthy in terms of objective variables. Additionally, in the case of TUNA, the length of the benefit is less than that observed among patients treated with TURP, with a significantly greater number [OR: 7.44 (2.47, 22.43)] of TUNA patients requiring new treatment for symptomatic BPH. It has to be recognized, however, that the TURP re-treatment rate found in this study is significantly lower than the figures previously reported in literature [2,59].

Insofar as safety is concerned and though the description of adverse effects was heterogeneous and in general ambiguous, pooled analysis of the literature shows that use of TUNA versus TURP entails a reduction in the absolute risk of complications about 19% with an estimated NNT of 5 to prevent a bad outcome. With regard to specific complications, the differences observed are particularly notable in terms of postoperative hemorrhage and sexual function disorders.

Although it has to be acknowledged that the body of evidence on which this review is based is of only moderate-low quality, its results indicate that while TUNA does not reach the same level of efficacy as TURP, it can provide adequate short-term symptom relief and improvement in quality of life with low postoperative morbidity. In this context, TUNA may be an attractive option for patients with a high symptom index but a low degree of obstruction who choose a surgical form of therapy and/or in those who wish to preserve their sexual function. Along the same lines, and given that TUNA has fewer anesthetic requirements than TURP, it may likewise be of special interest among patients with a high anesthetic risk or in those who do not wish to undergo general or regional anesthesia. Additionally, this study shows that TUNA is of short duration and generates a short hospital stay than TURP. Indeed, TUNA reduced the length of stay in hospital by two days. These data, albeit indirect, suggest that it could entail a reduction in costs vis-à-vis TURP therapy. There is no, however, good evidence supporting that TUNA registers a better cost-effectiveness ratio from a social perspective as none of the studies located conducted a formal cost-evaluation.

However, the usefulness of TUNA in symptomatic BPH must balance all these potential advantages against its high rate of re-treatment. This constitutes, in our opinion, the fundamental limitation of the technique given that duration of efficacy is a matter of vital importance to both patient and healthcare system owing to the personal and financial implications of the succession of treatments. In addition, the high secondary-treatment rate of TUNA should be taken into account both when analyzing if may entail a saving of healthcare resources against TURP.

As against other minimally invasive therapies, results differ vis-à-vis the comparator, with TUNA appearing as superior to some but similar or even inferior to others. However, analysis of available literature revealed a number of problems that hinder extrapolation of the results to clinical practice. Firstly, there is the fact that despite the extensive search performed, all studies found have a non-randomized design with multiple comparisons being established, something that tends to hamper evaluation. Secondly, some of the studies have a scant sample size, which renders assessment of results that much more difficult. Thirdly, half of them have an excessively short duration thereby being unable to appraise the long-tem efficacy of the techniques. Fourthly, there is an obvious lack of replication of comparisons, which not only prevented detailed evaluation of results but also discoloured the consistency of the evidence. All these factors make us concern about the current literature's capacity to provide the evidence necessary to determine the role of TUNA vis-à-vis other local forms of treatment for BPH and strongly support the need for better clinical research.

### Potential report limitations

It has to be recognized that publication bias is possible and that, by not including unpublished studies, the beneficial effects of the TUNA system may have been overestimated. Nevertheless, we feel that any such bias would necessarily have been minimized by the scope of and systematic strategy used in the search of the literature and are confident that majority of the research conducted in this field was successfully identified. So, in accordance with some recommendations [19,22] we have made a broad search of literature using at least three database sources supplemented by other search strategies. In addition, we have included both fully published studies and grey literature [60]. However, in line with prior reports we decided not to include unpublished data from industry given both the difficulties encountered in obtaining this information and the recognition that the use of these data may not necessarily reduce the bias in meta-analysis [60,61].

We also endeavored to reduce such biases by having the studies separately evaluated by two independent reviewers and using defined criteria for the purposes of study inclusion and analysis but, in accordance with some recent literature [60,62] we have not used funnel plots to examine the possibility of publication bias given the limitations and potential misleading results of these graphs.

The methodologic quality of the studies, and particularly the high percentage of non-controlled case-series could constitute another limitation of this study. This situation is particularly common in surgery [63], where less than 10% of the evidence sustaining surgical interventions comes from randomized studies [64] and where systematic reviews play an essential role in evaluating new technologies [65].

Although we are aware of the potential difficulties and limitations posed by such evidence, we completely agree with recent publications highlighting the usefulness of these studies for evaluating the efficacy and safety of therapeutic devices and procedures [7,63-69]. The evaluation of such studies in systematic reviews provides information on the possible benefits and risks of the procedures and on the limitations of the evidence supporting their use. Such studies moreover help identify those techniques that should be subjected to more rigorous evaluation, and identify areas of uncertainty which otherwise would not be addressed and which would allow some procedures to be accepted and others rejected in clinical practice, on the basis of individual appeal alone [65-68]. It is therefore not surprising that a large percentage of systematic reviews conducted by organizations of repute [63,68] include studies of case series, or that over 50% of all meta-analyses in the literature correspond to non-randomized studies [69]. Furthermore, the results of recent studies suggest that non-randomized and randomized trials may yield similar estimations of the effects of a given treatment [70] and that meta-analyses of non-randomized studies may produce results similar to those of meta-analyses of randomized clinical trials [21].

Here we have to say that we have tried to reduce the possible impact of the studies' design using a conservative random-effects model to combine the results of the individual reports in the meta-analysis [71], and that sensitivity analysis by excluding poorer quality studies in comparisons with TURP did not change our results. However, one of the main problems of the existing studies on TUNA is the absence of independent evaluation of the results obtained. To this we must also add that the number of losses was not reported in a fair number of studies. Consequently, it has to be recognized that detection and attrition biases may have influenced to some degree the results of the review [19,72].

A further problem could reside, in our opinion, in the fact that possible bias in the subjective variables might lead to the beneficial effects of TUNA being overestimated. In clinical drug trials, it has been observed that this effect is blocked by administering a course of placebo-based therapy to patients prior to the start of the study [73]. Yet, the studies included make no mention of such a measure having been taken, so that the influence of such a placebo effect on the results cannot be ruled out, an effect that is known to cause an improvement in symptoms and other functional parameters, such as Qmax, as well as clinically important adverse effects [73].

## Conclusion

Despite these limitations, the results of this review suggest that TUNA is a relatively effective and safe technique that may eventually prove to have a role in selected patients with symptomatic BPH. TUNA significantly improves BPH parameters with respect to baseline values, but it does not reach the same level of efficacy and long-lasting success as TURP. On the other hand, TUNA seems to be superior to TURP in terms of associated morbidity, anesthetic requirements and length of hospital stay. With respect to the role of TUNA vis-à-vis other minimally invasive forms of treatment, the results of this review indicate that there are insufficient data to define this with any degree of accuracy.

In addition, the review of the literature highlights the existence of a number of areas of uncertainty, chief among which, in our opinion, are: the precise mechanism of action of the technique and the factors implicated in the treatment's success; the lack of comparative studies with respect to medical treatment; the position of TUNA with respect to other minimally invasive therapies; and the overall cost-effectiveness ratio of the technique. Such data would be of critical importance to determine the exact role of TUNA in the treatment of symptomatic BPH.

### Implications for practice & policy

Apart from the need for the controlled clinical research and sound methodologic quality that would resolve the above-mentioned uncertainties, the following elements should be borne in mind before TUNA is sought to be introduced into routine clinical practice. Firstly, with regard to its indication, current evidence show that the potential advantages of TUNA in terms of morbidity and anesthetic requirements should be balanced with the fact that it does not reach the same level of efficacy and long-lasting success as TURP. Secondly, due to the fact that it has a greater impact on symptoms and less-pronounced impact on obstructive parameters, TUNA would appear to be especially indicated in the early stages of BPH. Nevertheless, the optimal point in the course of BPH progression for performing the technique has not yet been identified. Thirdly, account must be taken of the high re-treatment rate when the results of the procedure are presented in terms of use of healthcare resources.

## Competing interests

The author(s) declare that they have no competing interests.

## Authors' contributions

CB participated in the design and coordination of the study, carried out the critical appraisal of the included studies and assisted in the writing up. TL carried out the statistical analysis of studies and assisted in the writing up. AM and LN developed the literature search and assisted in the critical appraisal of included studies. JMA coordinated the project. All authors read and approved the final manuscript.

## Pre-publication history

The pre-publication history for this paper can be accessed here:



## Figures and Tables

**Table 1:** Summary of the characteristics of the non-comparative studies (n = 26)

**Characteristics**	**Mean (range)**	**Frequency studies/patients (%)**
**Sample size**	59 (range:10–206)	
**Age (years)**	67.5 ± 2.9 (47–90)	
**Follow-up (months) [n]**	16 ± 15 (1–63)	
**Continent **		
Europe		13 (50%)
America		6 (23%)
Asia		3 (11.5%)
Australia		3 (11.5%)
Africa		1 (4%)
**Studies in languages other than English**		3 (11.5%)
**Type of study **		
Design		
Prospective		24 (92%)
Retrospective		1 (4%)
Unknown		1 (4%)
		
Single-center		17 (65.4%)
Multicenter		9 (34.6%)
**Type of anesthesia **		
Local		3 (13%)
Local+sedation		10 (43.5%)
Spinal		3 (13%)
General		2 (8.7%)
General/local+sedation		5 (22%)
**Variables assessed**		
Symptoms		25 (96.2%)
Quality of life score		22 (84.6%)
Qmax (mL/s)		25 (96.2%)
PVR (mL)		17 (65.4%)
Sexual function		12 (46.2%)
Pressure-flow studies		7 (26.9%)
Prostatic size		13 (50%)
Re-treatment		17 (65.4%)
Adverse effects		22 (84.6%)
Duration of procedure		15 (57.7%)
Hospital stay		11 (42.3%)
**Quality assessment **		
Consecutive or representative sample		Yes: 21 No: 3 Not reported:2
Presence of cointerventions		Yes: 19 No: 1 Not reported:6
Evaluation blind or independent		Yes: 0 No: 26 Not reported:0
Losses to follow-up ≥ 20%		Yes:11 No:9 Not reported:6

**Table 2:** Efficacy of TUNA. Non-comparative studies

	**3 months**	**6 months**	**1 year**	**2 years**	**3 years**	**4 years**	**5 years**
*Symptom index*							

Studies	14^26,28–30,32,33,35,36,38,43,45–48^	14^25,26,28–30,32,33,35,36,38,39,41,43,45^	13^30,32–36,38,39,41,43–45, 47^	4^32,36,43,44^	2^42,44^	1^44^	2^44,49^
No. patients	650	661	868	403	297	206	394
Effect	-11.33	-12.52	-12.59	-12.98	-9.94	-10.2	-10.99
95% CI	-14.1,-8.6	-15, -9.9	-14, -11.4	-14.2,- 11.8	-10, -9	-11,-9.	-14,- 8.3
P-value	.000	.000	.000	.000	.000	.000	.000
%improvement	53%	59%	59%	61%	47%	48%	51%

*Quality-of-life score*							

Studies	12^26,28,30,32,33,35,36,38,43,45,47,48^	13^25,26,28,30,32,33,35,36,38,39,41,43,45^	14^30,32–36,38,39,41,43–47^	5^32,36,43,44,46^	1^44^	1^44^	2^44,49^
No. patients	545	646	959	496	206	206	394
Effect	-2.80	-2.79	-2.60	-2.51	-2.1	-1.9	-2.14
95% CI	-3.3, -2.3	-3.2,-2.4	-3, -2.15	-2.8, -2.23	‡	‡	-3.2, -1.
P-value	.000	.000	.000	.000	‡	‡	.000
%improvement	60%	60%	56%	54%	45%	41%	46%

*Qmax (mL/s)*							

Studies	15^25,26,28–30,33,35–38,43,45–48^	17^25,26,28–30,32–39,41,43,45,46^	14^30,32–36,38,39,41,43–47^	4^32,36,43,44^	2^42,44^	1^44^	2^44,49^
No. patients	578	817	960	403	297	206	394
Effect	5.381	5.32	4.40	5.029	3.48	4.10	3.53
95% CI	3.67,7.08	3.7, 6.9	2.94, 5.86	4.35, 5.7	1.5, 5.4	3.2, 4.9	2.9, 4.2
P-value	.000	.000	.000	.000	.001	.000	.000
%improvement	33%	34%	45%	37%	57%	49%	56%

*PVR (mL)*							

Studies	11^28,29,33,35–38,43,45,47,48^	10^28,29,33,35,36,38,39,43,45,46^	10^33–36,38,39,43,45,47^	2^45,52^	NA	NA	1^49^
No. patients	440	557	598	77			188
Effect	-29.55	-37.6	-32.10	-29.5			-58
95% CI	-38.9, -20	-49.7,-25.5	-44,-20	-47.5,- 11.5			-76,-40
P-value	.000	.000	.000	.001			.000
%improvement	28%	36%	31%	28%			56%

*P_*det *_at Qmax (cm H_*2*_O)*							

Studies	3^32,35,36^	7^26,29,30,32,35–37^	2^32,36^	2^32,36^	NA	NA	NA
No. patients	238	247	167	167			
Effect	-24.64	-25.36	-22.74	-24.48			
95% CI	-35.6,-13.6	-33.2,-17.5	-29.5, -16	-40, -8.92			
P-value	.000	.000	.000	.002			
%improvement	28%	29%	25%	28%			

*Prostatic size*							

Studies	7^26,28,31,33,35,43,48^	5^29,33,41,43,46^	4^30,33,43,46^	1^43^	NA	NA	1^49^
No. patients	242	257	140	8			131
Effect	-6.32	-6.45	-7.89	-7.9			1.9
95% CI	-9.1,-3.5	-9.6,-3.3	-10.6,-5.1	-17.7,1.9			-3.4,7.2
P-value	.00	.00	.00	.11			.48
% reduction	14%	14%	17%	17%			0%

Random effects model. Results are expressed as difference of means and percentage of improvement versus baseline. Abbreviations: CI denotes confidence interval. Qmax denotes maximum urinary flow rate. PVR: Post-void residual volume.‡ impossible to calculate due to lack of data. NA: not available

**Table 3:** Safety of TUNA. Non-comparative studies

**Adverse effect**	**No. events**	**Percentage of total patients**
Hematuria:		
- Mild	337	28%
- Moderate	85	7%
- Transitory unspecified severity	25	2%
- Severe	16	1%
Transitory urinary retention	279	23%
Dysuria	167	14%
Irritative symptoms	117	10%
Urinary tract infection	43	4%
Pain during procedure		
Moderate	11	0.9%
Intense	4	0.3%
Postoperative perineal pain	13	1%
Epidydimo-orchitis	11	0.9%
Mild-moderate burning sensation	10	0.8%
High fever	8	0.7%
Treatment halted due to intolerance to procedure	6	0.5%
Urethral stenosis	6	0.5%
Erectile dysfunction	4	0.3%
Hemospermia	4	0.3%
Retrograde ejaculation	3	0.2%
Loss of ejaculation	1	0.08%
Prostatism	2	0.2%
Complex bladder dysfunction	1	0.08%
Damage in the mucosa	1	0.08%
Deep vein thrombosis	1	0.08%

**Table 4:** Summary of the characteristics of studies that compare TUNA with other therapies

	**Author [reference number]**							
	**Bruskewitz [50]**	**Roehrborn [51]**	**Chandrasekar [52]**	**Cimentepe [53]**	**Hill [54]**	**Schatzl [55,57]**	**Arai [56]**	**Minardi [58]**

**Design **	RCT	RCT	RCT	RCT	RCT	Prospective cohort	Prospective cohort	Prospective cohort
**No. patients**	121	121	156	59	121	95	204	212
**TUNA [N. patients]**	65	65	76	26	65	15	51	24
**Comparator [No. Patients]**	TURP [56]	TURP [56]	TURP [76]	TURP [33]	TURP [56]	TURP [28] TUVP [17] VLAP [15] HIFU [20]	TURP [65] TUMT [40] ILC[48]	TURP [90] TUVP [13] ILC[71] WIT [13]
**Duration of symptoms**	> 3months	> 3months	Not reported	Not reported	> 3months	Not reported	Not reported	Not reported
**Anesthesia**	TUNA: Local TURP: General	TUNA: Local TURP: General	Not reported	Regional	TUNA: Local TURP: General	General/Regional	TUNA, ILC: Not reported TURP: Spinal TUMT: Local	TUNA-WIT:Local TUVP, ILC: Spinal TURP: General
**Variables assessed**	Symptom, QoL, Qmax, PVR, prostatic size, adverse effects duration of procedure, hospital stay	P_det_at Qmax No. Abrams-Griffiths	Symptom, QoL, Qmax, adverse effects, hospital stay, re-treatment	Symptom, QoL, Qmax, PVR, sexual function, prostatic size, adverse effects, duration of procedure, hospital stay, re-treatment	Symptom, QoL, Qmax, PVR, adverse effects, hospital stay, re-treatment	Symptom, QoL, Qmax, PVR, adverse effects, hospital stay, re-treatment	Symptom, QoL, Qmax, PVR, sexual function, adverse effects.	Symptom, QoL, Qmax, PVR, P_det _at Qmax, prostatic size, adverse effects, duration, re-treatment
**Follow-up**	12 months	6 months	7 years	18 months	5 years	6 weeks/24 m	3 months	24 months
**Randomization method specified **	Yes	Yes	No	No		Not applicable	Not applicable	Not applicable
**Inclusion/exclusion criteria specified**	Yes	Yes	No	Yes		Yes	Yes	Yes
**Concomitant treatment specified**	Yes	Yes	No	Yes		Yes	No	No
**Intention-to-treat analysis**	Yes	Yes	Not reported	Not reported		Yes	Not reported	Not reported
**Blind/Independent evaluation **	Not reported	Not reported	Not reported	Not reported		Not reported	Not reported	Not reported
**Losses**	TUNA:9% TURP:16%	None	Not reported	None	TUNA>72% TURP > 60%	TUVP [4] VLAP [4] HIFU [4] TUNA [3]	TUNA [9] TURP [10] TUMT [6], ILC [6]	Not reported

Abbreviations: RCT: Randomized controlled trial. TUNA (Transurethral Needle Ablation). TURP (Transurethral resection). TUMT (Transurethral Microwave Thermotherapy). WIT (Water-induced Thermotherapy). ILC (Interstitial Laser Coagulation). TUVP (Transurethral Electrovaporization of the Prostate). VLAP (Visual Laser Ablation of the Prostate). HIFU (Transrectal High Intensity Focused Ultrasound). QoL: Quality of life score. Qmax: Maximum urinary flow rate. PVR: Post-void residual volume. Pdet at Qmax: Detrusor presures at peak flow rate.

**Table 5:** TUNA vs. TURP: results of meta-analysis of variables of efficacy.

Variable	Studies (No. patients)	**TUNA** Difference in means (% improvement)	**TURP** Difference in means (% improvement)	**TUNA vs. TURP** Difference in means (95% CI), P value
*Symptom score*				

3 months	3^50,53,56 ^(384)	-12.49 (57%)	-13.85 (64 %)	1.33 (-.81,3.47), .22
1 year	4^52–54,57 ^(427)	-12.09 (55%)	-15.48 (71%)	3.69 (2.09, 5.29), .00
2 years	3^52,54,57 ^(368)	-10.42 (48%)	-14.83 (68%)	4.72 (3.77, 5.69), .00
3 years	2^52,54 ^(273)	-9.83 (45%)	-14.63 (67%)	4.82 (4.25, 5.39), .00
4 years	1^54 ^(121)	-10.80 (50%)	-16.50 (76%)	5.70 (4.67, 6.73), .00
5 years	1^54 ^(121)	-13.30 (61%)	-13.30 (61%)	0 (-0.99, 0.99), 1.0

*Quality of life score*				

3 months	3^50,53,56 ^(277)	-4.47 (54%)	-3.65 (47%)	-0.40 (-1.25, 0.45), .35
1 year	3^52–54 ^(332)	-4.05 (49%)	-4.34 (56%)	0.63 (0.12, 1.15), .01
2 years	2^52,54 ^(273)	-3.90 (47%)	-5.67 (74%)	1.69 (-1.05, 4.44), .22
3 years	2^52,54 ^(273)	-4.17 (50%)	-5.18 (67%)	1.02 (0.14, 1.90), .02
4 years	1^54 ^(121)	-6.6 (80%)	-8.90 (116%)	2.3 (1.84, 2.76), .00
5 years	1^54 ^(121)	-8.0 (96%)	-8.60 (112%)	0.6 (0.21, 0.99), .00

*Qmax (mL/s)*				

3 months	3^50,53,56 ^(277)	4.85 (57%)	11.37 (148%)	-6.48 (-7.29, -5.68), .00
1 year	4^52–54,57 ^(375)	6.49 (76%)	12.23 (160%)	-5.9 (-7.73, -4.08), 00
2 years	4^50,54,57,58 ^(430)	4.55 (53%)	12.17 (159%)	-7.93 (-11.1, -4.77), .00
3 years	2^52,54 ^(273)	5.6 (66%)	10.80 (141%)	-5.32 (-6.78, -3.85), .00
4 years	1^54 ^(121)	2.9 (34%)	10.10 (132%)	-7.2 (-7.99, -6.4), .00
5 years	1^54 ^(121)	2.6 (30%)	9.8 (127%)	-7.2 (-7.93, -6.47), .00

*PVR (ml)*				

3 months	3^50,53,56 ^(277)	-27.76 (31%)	-44.66 (44%)	23.82 (-19.35, 66.97), .28
1 year	3^53,54,57 ^(223)	-21.92 (24%)	-52.42 (51%)	24.97 (20.90, 29.05), .00
2 years	3^54,57,58 ^(278)	-27.58 (31%)	-69.24 (68%)	35.8 (25.00, 46.61), .00
5 years	1^54 ^(121)	-31.40 (35%)	-54.50 (53%)	23.1 (12.16, 43.04), .00

*PdetQmax (cm of water)*				

6 months	2^51,58 ^(235)	-20.52 (27%)	-32.4 (45%)	13.12 (0.6, 25.65), .04
2 years	1^58 ^(114)	-28.56 (38%)	-42 (58%)	13.44 (11.6,15.27), .00

*No. Abrams-Griffiths*				

6 months	1^51 ^(121)	-24 (39%)	-47.40 (81%)	23.4 (20.7, 26.2), .00

*Prostatic size*				

3 months	2^50,58 ^(235)	-2.66 (7%)	-12.98 (34%)	10.13 (-7.02,27.3), .02
1 year	1^50 ^(121)	-0.9 (2.4%)	-5.5 (17%)	6.4 (4.8,7.96), .00
2 years	2^53,58 ^(173)	-6.46 (15%)	-12.78 (34%)	5.52 (-2.7,13.75), .19

Random effects model. Results are expressed as difference of means and percentage of improvement versus baseline. Abbreviations: CI denotes confidence interval. Qmax denotes maximum urinary flow rate. PVR: Post-void residual volume.

**Table 6:** TUNA vs. other minimally invasive therapies. Efficacy

Variable	No. studies (patients)	**TUNA** Difference in means (% improvement)	**TUMT** Difference in means(% improvement)	**TUNA vs. TUMT **WMD (95% CI), P value
*Symptom score*				

Pre-3 months	1^56 ^(76)	-9.3 (48%)	-5.2 (28%)	-4.1 (-5.02, -3.18), .00

*Quality-of-life score*				

Pre-3 months	1^56 ^(76)	-2.3 (49%)	-1.7 (39%)	-0.6 (-0.75, -0.44), .00

*Qmax (mL/s)*				

Pre- 3 months	1^56 ^(76)	1 (11%)	0.9 (12%)	0.1 (-0.47, 0.67), .73

Variable	No. studies (patients)	**TUNA ** Difference in means (% improvement)	**WIT ** Difference in means (% improvement)	**TUNA vs. WIT ** WMD (95% CI), P value

*Qmax (ml/sec*				

Pre- 6 months	1^58 ^(37)	3.2 (41%)	1.8 (14%)	1.4 (-0.13, 2.93), .07
Pre- 2 years	1^58 ^(37)	4.9 (44%)	2.2 (18%)	2.7 (1.14, 4.26), .00

*PVR (mL)*				

Pre- 6 months	1^58 ^(37)	-58.3 (62%)	-18.5 (25%)	-39.8 (-46.4, -33.2), .00
Pre- 2 years	1^58 ^(37)	-40.9 (54%)	-23.3 (49%)	-17.6 (-24, -11.2), .00

*P_*det *_at Qmax (cm H_*2*_O)*				

Pre- 6 months	1^58 ^(37)	-22.52 (35%)	-17.5 (27%)	-5.02 (-9.36, -0.65), .02
Pre- 2 years	1^58 ^(37)	-28.56 (44%)	-15.7 (25%)	-12.9 (-17.4, -8.34), .00

Variable	No. studies (patients)	**TUNA ** Difference in means (% improvement)	**ILC ** Difference in means (% improvement)	**TUNA vs. ILC ** WMD (95% CI), P value

*Symptom score*				

Pre- 3 months	1^56 ^(84)	-9.3 (48%)	-12.4 (64%)	3.1 (2.36, 3.84), .00

*Quality-of-life score*				

Pre- 3 months	1^56 ^(84)	-2.3 (49%)	-2.5 (58%)	0.2 (0.06, 0.34), .00

*Qmax (mL/s)*				

Pre- 3 months	1^56 ^(84)	1 (11%)	5 (53%)	-4 (-4.64, -3.36), .00
Pre- 6 months	1^58 ^(95)	3.2 (41%)	2.8 (30%)	0.4 (-0.15, 0.95), .15
Pre- 2 years	1^58 ^(95)	4.9 (44%)	3 (32%)	1.9 (1.27,2.52), .00

*PVR (mL)*				

Pre- 3 months	1^56 ^(84)	-22.1 (26%)	-71.9 (72%)	49.8 (41.99, 57.60), .00
Pre- 6 months	1^58 ^(95)	-58 (62%)	-45 (45%)	-13 (-16.41, -10.2), .00
Pre- 2 years	1^58 ^(95)	-41 (54%)	-42 (42%)	1.3 (-2.07, 4.67), .45

*P_*det *_at Qmax (cm H_*2*_O)*				

Pre- 6 months	1^58 ^(95)	-22.5 (35%)	-25.8 (38%)	3.28 (1.4, 5.16), .00
Pre- 2 years	1^58 ^(95)	-28.6 (44%)	-23 (34%)	-5.56 (-7.76, -3.36), .00

Variable	No. studies (patients)	**TUNA ** Difference in means (% improvement)	**TUVP ** Difference in means (% improvement)	**TUNA vs. TUVP ** WMD (95% CI), P value

*Symptom score*				

Pre- 6 months	1^57 ^(32)	-9 (47%)	-13.1 (69%)	4.1 (2.38, 5.82), .00
Pre-1 year	1^57 ^(32)	-11.2 (58%)	-13.3 (70%)	2.1 (0.72, 3.48), .00
Pre- 2 years	1^57 ^(32)	-10 (52%)	-12.7 (66%)	2.7 (1.13, 4.27), .00

*Qmax (mL/s)*				

Pre- 6 months	2^57,58 ^(69)^a^	3.42 (41%)	10.25 (108%)	-6.08 (-8.6, -3.5), .00
Pre- 1 year	1^57 ^(32)	2.6 (30%)	12.4 (132%)	-9.8 (-10.97, -8.63), .00
Pre- 2 years	2^57,58^(69)^b^	2.76 (44%)	8.93 (97%)	-6.1 (-11.45, -0.67), .00

*PVR (mL)*				

Pre- 6 months	2^57,58 ^(69)^c^	-57.26 (62%)	-61.84 (76%)	10.96 (0.26, 21.65), .04
Pre- 1 year	1^57 ^(32)	-53 (62%)	-51 (67%)	-2 (-16.15, 12.15), .78
Pre- 2 years	2^57,58 ^(69)^b^	-42.06 (54%)	-61.20 (74%)	10.87 (-20.54, 42.3), .49

*P_*det *_at Qmax (cm H_*2*_O)*				

Pre- 6 months	1^58 ^(37)	-22.52 (35%)	-40 (53%)	17.48 (13.8, 21.2), .00
Pre- 2 years	1^58^(37)	-28.56 (44%)	-35 (47%)	6.44 (2.32, 10.56), .00

Variable	No. studies (patients)	**TUNA ** Difference in means (% improvement)	**VLAP ** Difference in means (% improvement)	**TUNA vs. VLAP ** WMD (95% CI), P value

Symptom score				

Pre- 6 months	1^57 ^(30)	-9 (47%)	-7.3 (37%)	-1.7 (-4.0,0.62), .15
Pre-1 year	1^57 ^(30)	-11.2 (58%)	-10.8 (55%)	-0.4 (-2.48, 1.68), .70
Pre- 2 years	1^57 ^(30)	-10 (52%)	-12.9 (65%)	2.9 (1.3, 4.5), .00

*Qmax (mL/s)*				

Pre- 6 months	1^57 ^(30)	4.3 (41%)	8.6 (141%)	-4.3 (-6.17, -2.43),.00
Pre- 1 year	1^57 ^(30)	2.6 (30%)	7.8 (128%)	-5.2 (-6.3, -3.57), .00
Pre- 2 years	1^57 ^(30)	2.3 (44%)	5.6 (92%)	-3.3 (-4.7, -1.8), .00

*PVR (mL)*				

Pre- 6 months	1^57 ^(30)	-44 (62%)	-63 (67%)	19 (1.90, 36.1), .02
Pre- 1 year	1^57 ^(30)	-53 (62%)	-64 (68%)	11 (-6.44, 28.44), .2
Pre- 2 years	1^57 ^(30)	-55 (54%)	-68 (72%)	13 (-5.6, 31.6), .17

Variable	No. studies (patients)	**TUNA ** Difference in means (% improvement)	**HIFU ** Difference in means (% improvement)	**TUNA vs. HIFU** WMD (95% CI), P value

Symptom score				

Pre- 6 months	1^57 ^(35)	-9 (47%)	-8.3 (56%)	-0.7 (-2.35, 0.95), .40
Pre- 1 year	1^57 ^(35)	-11.2 (58%)	-10.4 (71%)	-0.8 (-2.11, 0.51), .23
Pre- 2 years	1^57 ^(35)	-10 (52%)	-7 (48%)	-3.0 (-4.71, -1.29), .00

*Qmax (mL/s)*				

Pre- 6 months	1^57 ^(35)	4.3 (41%)	3.9 (42%)	0.40 (-9.4, 1.74), .55
Pre- 1 year	1^57 ^(35)	2.6 (30%)	3.9 (42%)	-1.30 (-2.16, -0.44), .00
Pre- 2 years	1^57 ^(35)	2.3 (44%)	2 (22%)	0.30 (-0.52, 1.12), .47

PVR (mL)				

Pre- 6 months	1^57 ^(35)	-44 (62%)	-48 (51%)	4.00(-10.44, 18.44), .58
Pre- 1 years	1^57 ^(35)	-53 (62%)	-45 (48%)	-8.00 (-22.44, 6.44), .27
Pre- 2 years	1^57 ^(35)	-55 (54%)	-36 (38%)	-19 (33.69, -4.31), .01

Abbreviations. TUMT: Transurethral Microwave Thermotherapy. WIT: Water-induced Thermotherapy. ILC: Interstitial Laser Coagulation. TUVP: Transurethral Electrovaporization of the Prostate. VLAP: Visual Laser Ablation of the Prostate. HIFU: Transrectal High Intensity Focused Ultrasound.

WMD: Weighted mean difference. Qmax: Maximum urinary flow rate. PVR: Post-void residual volume. Pdet at Qmax: Detrusor presures at peak flow rate.

**Table 7:** TUNA vs. other Minimally Invasive Therapies: adverse effects

	**TUNA**	**TUMT**	**ILC**	**TUVP**	**VLAP**	**HIFU **	**WIT**
Erectile dysfunction	7 (20%)	6 (26%)	7 (18%)	--	--	--	--
Epidydimitis	0 (0%)	--	--	0 (0%)	--	--	--
Urethral stenosis	--	--	--	2 (15%)	--	--	--
Retrograde ejaculation	9 (14%)	--	16(15%)	12 (92%)	--	--	--
Fever	--	--	1(1.4%)	1 (7.7%)	--	--	--
Transitory incontinence	--	--	--	2 (15%)	--	--	--
Urinary infection	--	--	--	2 (15%)	--	--	--
Urinary retention	--	--	--	1 (5.9%)	--	--	3 (24%)
Rehospitalization	1 (6.7%)	--	--	1 (5.9%)	1 (7%)	1 (5%)	4 (30.7%)
Irritative symptoms	--	--	30 (42%)	--	--	--	--
Reduction in seminal volume	--	--	15 (21%)	--	--	--	--
Hematuria	1 (6.8%)	--	25 (35.2%)	2 (11.8%)	0 (0%)	1 (5%)	--
Dysuria	2 (8.3%)	--	--	--	--	--	--
Change in sexual activity	0 (0%)	--	--	--	--	--	--

Results are expressed as number of cases and percentages. Abbreviations. TUMT: Transurethral Microwave Thermotherapy. WIT: Water-induced Thermotherapy. ILC: Interstitial laser coagulation TUVP: Transurethral Electrovaporization of the prostate. VLAP: Visual Laser Ablation of the prostate. HIFU: Transrectal High Intensity Focused Ultrasound.
